# A Current Review of Water Pollutants in American Continent: Trends and Perspectives in Detection, Health Risks, and Treatment Technologies

**DOI:** 10.3390/ijerph20054499

**Published:** 2023-03-03

**Authors:** Walter M. Warren-Vega, Armando Campos-Rodríguez, Ana I. Zárate-Guzmán, Luis A. Romero-Cano

**Affiliations:** Grupo de Investigación en Materiales y Fenómenos de Superficie, Facultad de Ciencias Químicas, Universidad Autónoma de Guadalajara, Av. Patria 1201, Zapopan C.P. 45129, Jalisco, Mexico

**Keywords:** wastewater treatment, environmental public health, environmental impact assessment, inorganic pollutants, organic contaminants

## Abstract

Currently, water pollution represents a serious environmental threat, causing an impact not only to fauna and flora but also to human health. Among these pollutants, inorganic and organic pollutants are predominantly important representing high toxicity and persistence and being difficult to treat using current methodologies. For this reason, several research groups are searching for strategies to detect and remedy contaminated water bodies and effluents. Due to the above, a current review of the state of the situation has been carried out. The results obtained show that in the American continent a high diversity of contaminants is present in the water bodies affecting several aspects, in which in some cases, there exists alternatives to realize the remediation of contaminated water. It is concluded that the actual challenge is to establish sanitation measures at the local level based on the specific needs of the geographical area of interest. Therefore, water treatment plants must be designed according to the contaminants present in the water of the region and tailored to the needs of the population of interest.

## 1. Introduction

Water contamination represents a current crisis in human and environmental health. The presence of contaminants in the water and the lack of basic sanitation hinder the eradication of extreme poverty and diseases in the poorest countries [1]. For example, water sanitation deficiency is one of the leading causes of mortality in several countries. Due to unsafe water and a lack of sanitation, there are several diseases present in the population [2,3,4]. Therefore, the sixth global objective of the United Nations, foreseen as part of its sustainable development agent 2030, aims to guarantee the availability and sustainable management of water resources. In this sense, numerous research groups have focused on proposing alternative solutions focusing on three fundamental aspects: (a) detection of contaminants present in water for human consumption, (b) assessment of risks to public and environmental health due to the presence of contaminants in the water, and (c) the proposal of water treatment technologies. In the case of the American continent, the detection of contaminants (inorganic and organic) has been studied; the research works show alarming results in which the impact of water pollution is demonstrated, how the ecosystem is being affected, and consequently the repercussion towards human health [5,6,7,8]. This last point becomes worrying due to the fact that there are reported cases in which newborns, children, and adults consumed drinking water from various sources (such as rivers, lakes, groundwater, and wells) without the certainty that it is free of contaminants, representing a health risk factor [9,10,11]. Some of the detected contaminants have been associated with a potential health risk, such as the case of some disinfectants with cancer [12] and NO_3_^−^ and NO_2_^−^ as potential carcinogens in the digestive system [13]. The lack of safe drinking water has been reported in several countries [3,14] since the presence of contaminants in water has demonstrated that actual quality controls are not able to detect or treat pollutants that are present [15,16,17,18].

In this sense, numerous research groups have focused on proposing alternative solutions focusing on three fundamental aspects: (a) the detection of contaminants present in water for human consumption, (b) assessment of risks to public and environmental health due to the presence of contaminants in the water, and (c) a proposal for water treatment technologies. This communication shows a critical review of the latest published research works. The use of Web Of Science from Clarivate Analytics was used for the bibliographic review. The bibliographic search was carried out in January 2023 using the keywords “public health pollutants/contaminants water” + “name of the American country.” The retrieved articles were filtered considering the following: (i) articles published in the period 2018–2023, (ii) articles carried out based on effluents and bodies of water belonging to the American continent, and (iii) articles that demonstrate the presence and/or treatment of organic (excluding biological contaminants) and inorganic contaminants in water. The selection of these research articles was used to carry out a critical review of the current situation to propose future challenges to achieve efficient, and sustainable water treatment processes.

## 2. Critical Review: Evaluation of the Current Situation, Perspectives, and Challenges in the Detection of Contaminants, Health Risk Assessment, and Water Treatment Technologies in the American Continent

### 2.1. Detection of Contaminants in Water

At present, there are various analytical techniques that have been used in the detection and quantification of inorganic and organic contaminants in aqueous matrices. Mainly, these techniques can be divided into three major groups: chromatographic, spectroscopic, and other techniques, such as electrochemical and colorimetric titration. A comparison of the advantages and disadvantages of the most commonly used analytical techniques is presented in Supplementary Table S1. From these, techniques that have been used the most are shown below.

In chromatographic techniques, the most reported are gas chromatography-mass spectrometry (GC-MS), gas chromatography/mass spectrometry with selected ion monitoring (GC-MS/SIM), liquid chromatography-mass spectrometry (LC-MS), liquid chromatography quadrupole time-of-flight- mass spectrometry (LC-QTOF-MS), high-performance liquid chromatography-electrospray ionization-mass spectrometry (HPLC-ESI-MS), ultra-performance liquid chromatography- electrospray ionization-mass spectrometry (UPLC-ESI-MS), high-performance liquid chromatography-charged aerosol detector (HPLC-CAD), and ion chromatography (IC). In the case of spectroscopic techniques, these include inductively coupled plasma mass spectrometry (ICP-MS), atomic absorption spectrometry (AAS), inductively coupled plasma dynamic reaction cell mass spectrometry (ICP-DRC-MS), thermal ionization mass spectrometry (TIMS), high resolution inductively coupled plasma mass spectrometry (HR-ICP-MS), particle-induced X-ray emission (PIXE), fluorescence spectrometry, inductively coupled optical emission spectrometry (ICP-OES), and cold vapor atomic absorption spectrophotometry (CVAAS).

From these techniques, it has been possible to determine the concentrations of various pollutants of interest to human health and the environment.

**Figure 1:**
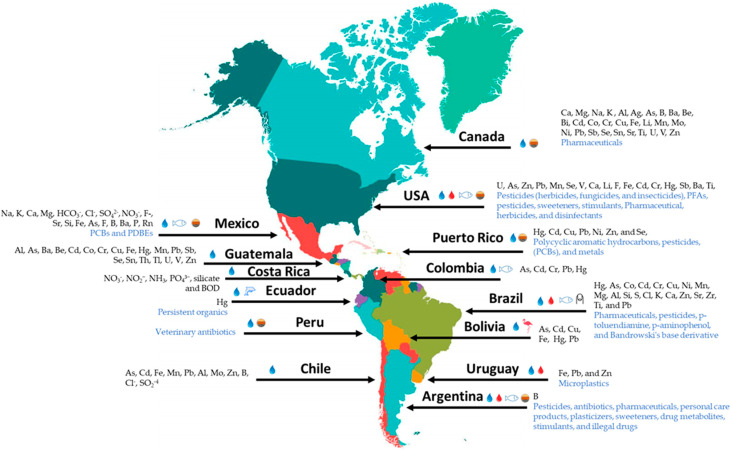
Geographical distribution of pollutants detected in the American continent in different matrices (water, blood, sediments, biota) in the last 5 years.

The compilation of information from the latest scientific reports (related to the detection of inorganic contaminants present in the water) is shown in Table 1 and Figure 1 (geographical distribution). On the other hand, the comparison of the detection limits for the limits of interest using different analytical techniques is presented in Supplementary Table S2. Among them, some works have been carried out based on water bodies in different countries, such as Canada [19], USA [20,21,22], Mexico [23], and Brazil [24], in which the presence of As, Fe, U, Zn, Na, K, Ca, Mg, HCO_3_^−^, and Hg with respect to interactions among water, bedrock mineralogy, and geochemical conditions of the region has been studied, so they can be classified as contamination due to a natural source. A particular case can be analyzed for U, which is present in water bodies of the southwest and west central USA, because high levels of acute exposure can be fatal for the population, and chronic exposure at low levels is associated with health problems, such as renal and cardiac risk. Although, exposure studies of surrounding communities cannot be considered conclusive, they correspond to a great advance in the field, and future studies should be carried out to assess possible damage to human health and the ecosystem.

On the other hand, research works stand out showing that water pollution can occur due to anthropogenic activities [25], being evident that modern practices of agriculture and livestock have consequences as the indiscriminate use of fertilizers, pesticides, and hormones results in nitrates in the water, which are associated with a risk of congenital anomalies, such as heart and neural tube defects.

Within the works carried out, one of the most concurrent techniques used in the evaluation of contaminants has been performed via ICP (MS or OES) due to its high precision, low cost, low detection limits, and the advantage of analyzing a large number of elements simultaneously in a short time [26]. However, in some cases, the detection limits of the technique are above the maximum permissible limits proposed by the WHO (World Health Organization), such is the case of Hg, for which the detection limit is of 0.0025 mg L^−^^1^ and the maximum detection limit recommended by the WHO is 0.002 mg L^−^^1^. Therefore, it is concluded that one of the challenges to be dealt with for metal detection in water is based in the fact that current techniques must be complemented by advanced analytical techniques, such as electrochemical tests [27]. These techniques are of great interest for their study due to the benefits they have, such as improvements in detection limits, low operating costs, short analysis times, and mobility, being able to perform analytical determinations in situ [27]. It is concluded that the contaminants with the greatest presence in the continent are As, U, Pb, Mn, Se, and Hg, mainly related to the mineralogy of the analyzed site and anthropogenic activities in the analysis areas. However, in some cases, the source of contamination is natural and occurs periodically due to seasonal changes, with the rainy season being the period with the greatest presence due to the mobility of metals contained in the rock and soil of the region [28,29]. Moreover, the presence of ions in solution related to the use of fertilizers and agrochemicals in crop fields has also been documented [30]. It is important to denote that the origin of the contamination source is not accurately concluded, providing a current challenge for the exact determination of the source to propose containment and sanitation actions to solve the problem.

**Table 1:** Detection of inorganic pollutants in environmental samples.

Analyte	Samples	Region	Environmental Risk Assessment	Analytical Technique	Ref.
As, Mn, Fe, CaCO_3_	Well water	Western Quebec (Canada)	Potential neuronal damage	ICP-MS	[19]
U, As, Zn	Well water	South-central Montana (USA)	Carcinogenic risk	ICP-MS	[22]
As, U, Pb, Mn, Se	Groundwater	Arizona, New Mexico, and Utah (USA)	Decreased cognitive function, cardiovascular and renal problems, neurotoxicity	ICP-OES	[21]
Na, K, Ca, Mg, HCO_3_^−^, Cl^−^, SO_4_^2−^, NO_3_^−^, F^−^, Sr, Si, Fe	Groundwater	Arid US–Mexican border Tecate, Baja California (Mexico)	Not mentioned	Multimeter, titration, ICP-MS, chromatography	[23]
As	Well water	Nova Scotia (Canada)	Risk of bladder and kidney caner	ICP-MS	[31]
V, Ca, As, Mn, Li, and U	Groundwater	Navajo Nation (USA)	Potential neuronal damage and carcinogenic risk	ICP-MS and ICP-OES	[20]
Hg	River fish	Western Amazon Basin (Brazil)	Risk of mercurialism	Cold vapor atomic absorption spectrophotometry	[24]
Pb	Surface and groundwater	Eastern half of USA and California (USA)	Adverse health effects in humans (ingested, inhaled, or imbedded)	TIMS HR-ICP-MS	[32]
Alkalinity (as CaCO_3_), SO_4_^2^^−^, Cl^−^, NO_3_^−^, Br^−^, F^−^, Inorganic phosphorus, total dissolved sulfide, Ca, Mg, Na, K, Al, Ag, As, B, Ba, Be, Bi, Cd, Co, Cr, Cu, Fe, Li, Mn, Mo, Ni, Pb, Sb, Se, Sn, Sr, Ti, U, V, Zn	Groundwater	Quebec (Canada)	Not mentioned, but is of public health concern	Titration and colorimetric methods ICP-MS IC	[33]
F^−^	Groundwater	USA	Multiple adverse human health effects	Not specified	[34]
Temperature, salinity, dissolved oxygen, chlorophyll, NO_3_^−^, NO_2_^−^, NH_3_, PO₄^3^^−^, silicate and BOD	Sea water	Gulf of Papagayo, North Pacific	Not mentioned	Spectrophotometric techniques	[35]
As, Cd, Cr, Cu, Ni, Mn, and Pb	Surface water	Joanes River, (Brazil)	Little or no health risk	ICP-MS	[28]
NO_3_^−^	Drinking water	California (USA)	Association with risk of spontaneous preterm birth	Historical data	[36]
As	Well water	USA	Future research to assess arsenic exposure with health outcomes	Historical data	[37]
Ti	Squid, swimming crabs, and shrimp	Brazil	Potential health risk	ICP-MS	[38]
As, Fe, Li, Mn, Mo, Pb, and U.	Well water	Nevada, (USA)	Negative health effects	ICP-MS	[39]
As, Cd, Pb, Mn, Hg, Cr	Well water	North Carolina (USA)	Potential health risk	Historical data	[40]
As, Na, K, Ca, Mg, Li, B, Fe, As, Ba, P, Rn, Si, S, Cl^−^, Br^−^, NO_3_^−^, SO_4_^2^^−^, F^−^ Water isotopes (the ratios of δ^18^O and δ^2^H)	Well water	Guanajuato (Mexico)	Health risk (carcinogen)	Titration methods ICP-MS Picarro cavity ring-down system IC	[41]
As, Cd, Fe, Mn, Pb, Al, Mo, Zn, B, Cl^−^, SO_2_, pH, electrical conductivity, and %Na	Surface water	Altiplano-Puna (Chile)	Potential human health risk	Mathematical models	[42]
Mn, Cr, Cu, Mg, Al, Si, P, S, Cl, K, Ca, Ti, Fe, Ni, Zn, Sr and Zr	Surface water	Rio Grande do Sul (Brazil)	Genotoxic and mutagenic effects in cell assays	PIXE	[43]
Sb, As, Ba, Be, Cd, Cr, Hg, Se, Tl and U	Surface water	USA	Potential human health risk	Historical data	[44]
Al, As, Ba, Be, Cd, Co, Cr, Cu, Fe, Hg, Mn, Pb, Sb, Se, Sn, Th, Tl, U, V, Zn	Tap water	Guatemala City (Guatemala)	Potential human health risk	ICP-MS	[45]
Al, As, Ba, Cd, Co, Cr, Cu, Fe, Ni, Pb, Se, and Zn	Canned Sardines	Brazil	Potential human health risk	ICP-OES	[46]
As and F	Ground water	Durango (Mexico)	Potential human health risk	Historical data	[47]
As	Groundwater	Comarca Lagunera (Mexico)	Potential human health risk	Historical data	[48]
Cr, Pb, and Hg	Seawater and fish	Gulf of Urabá (Colombia)	Potential human health risk	MIP-OES	[49]
As, Cd, Cu, Fe, Hg, Pb, and Zn	Water, sediment, Flamingo eggshells, feathers, and blood	Lake Uru Uru (Bolivia)	Potential human and wildlife health	Graphite furnace AA, Atomic fluorescence	[50]
As	Groundwater, surface water, and rainwater-harvesting tanks	Lake Poopó (Bolivia)	Potential human health risk	AAS, semiquantitative modified Gutzeit-method field asrsenic kit	[51]
Hg, As, Cd, and Pb	Eight fish species	Atrato River Delta, Gulf of Urabá (Colombia)	Potential human health risk	MIP-OES	[52]

Research studies presented in Table 1 demonstrated the potential human health risks that metal presence can have in water bodies, being important to highlight that there is still a need to evaluate the impact that inorganic contaminants have on human health. Furthermore, several research groups in different countries have detected the presence of contaminants not only in the supply sources, such as water bodies, but also in aquatic environments, such as flora and fauna being affected and representing economic importance since certain species can be traded, based on great demand to satisfy local and international markets.

On the other hand, organic contaminants can be divided into several groups; nevertheless, the principal groups are the ones denominated as persistent organic pollutants (POPs). These pollutants have an important impact on the environment and human health. Some examples are per- and polyfluoroalkyl substances (PFAS), personal care products, pharmaceutical compounds, pesticides, phenolic compounds, dyes, hormones, sweeteners, surfactants, and others.

Their detection has been primarily necessary to assess the effects that these pollutants have. Most of them are primarily obtained from industrial activities having different uses, such as flame retardants, coolants, cement, and others. Their presence represents an important contribution to water ecotoxicity (Ecuador, Argentina, Mexico) that affects the integrity of the species that inhabit that ecosystem [53,54,55].

Important issues have been detected in aquatic environments. The bioaccumulation of several organic compounds, such as polychlorinated biphenyl compounds (PBCs) and polybrominated diphenyl ethers (PBDEs), in important water bodies, such as Lake Chapala (Mexico), has been reported, through the analysis of samples recollected from water, fish, and sediments from two local seasonal periods. In this case, the fish analyzed were *Cyprinus carpio*, *Oreochromis aureus*, and *Chirostoma* spp., establishing that these chemical substances can reach the lake via industrial activities and strong winds and enter from the Lerma River (Mexico) [55].

In the study of Ramos et al. (2021), a water analysis was performed in the river and its treated water throughout a year in Minas-Gerais (Brazil). The detection of seventeen phenolic compounds with a single quadrupole gas chromatograph-mass spectrometer equipment (GCMS-QP2010 SE) coupled with a flame ionization detector (FID) was analyzed. From the samples analyzed, only sixteen were detected, being that 3-methylphenol was the only one not detected. In raw water, the detection of 2,3,4-trichlorophenol, 2,4-dimethylphenol, and 4-nitrophenol was found with the most frequency and for treated water, 4-nitrophenol and bisphenol A, establishing that a health risk to the environment and humans was identified with the contamination of these phenolic compounds [56]. Another study carried out in the St. Lawrence River, Quebec, (Canada), was performed based on an analysis of surface water for the detection of ultraviolet absorbents (UVAs) and industrial antioxidants (IAs). The detection was carried out via gas chromatography-mass spectrometry (GC-MS) detecting several groups of UVAs, such as organic UV filters (benzophenone (BP), 2-ethylhexyl salicylate (EHS), 2-hydroxy-4-methoxybenzophenone (BP3), 3,3,5-trimethylcyclohexylsalicylate (HMS), 2-ethylhexyl 2-cyano-3,3-diphenylacrylate (OC), and ethylhexyl methoxycinnamate (EHMC)), aromatic secondary amines (diphenylamine (DPA)), benzotriazole UV stabilizers (2-(2H-benzotriazol-2-yl)-4,6-di-tert-pentylphenol (UV238), and synthetic phenolic antioxidants (2,6-di-tert-butyl-4-methylphenol (BHT) and 2,6-di-tert-butyl-1,4-benzoquinone (BHTQ)). The field-based tissue-specific bioaccumulation factors (BAF) were analyzed to assess these contaminants in fish tissues (lake sturgeon and northern pike) in which some of the compounds that accumulated in lake sturgeon were BP3, BHT, and UV238. For northern pike, some were BP, BP3, BHT, and BHTQ, establishing an environmental risk assessment in terms of possible adverse effects on fish [57].

Finally, in the case of PAHs, several compounds have been detected (fluorene, naphthalene, anthracene, chrysene, and others) in different American countries, such as Canada, United States of America, Ecuador, Peru, Chile, and Brazil [58,59,60,61,62,63,64,65,66]. Their presence has been related to anthropogenic activities, such as aluminum smelter or oil production, having a negative impact on health, such as carcinogenic effects.

For this reason, analytical assays must be performed to establish the concentrations of these pollutants using techniques that are capable of studying a complex matrix and if it is possible, in situ. In Table 2, the description of several studies that were able to detect organic compounds in environmental samples and the technique that was employed are provided.

As it can be appreciated in Table 2, a variety of organic compounds have been identified as being associated with several disorders and diseases. Nevertheless, most of the studies analyzed correlated its contaminant of interest with previous research that evaluated its potential human health risk effect. For this reason, it is important to detect the contaminant and correlate it with its health impact in the environment (population and biota).

### 2.2. Presence of Pollutants in Water: Impact on Human Health and Its Possible Sources

The inorganic contaminants with the greatest presence in water bodies correspond to heavy metals. At the moment, the potential damage to health due to heavy metals has been reported as listed below: As(III) (skin damage, circulatory system issues), Cd(II) (kidney damage, carcinogenic, cardiovascular damage, hematological, and skeletal changes), Cr(III) (allergic dermatitis, diarrhea, nausea, and vomiting), Cu(II) (gastrointestinal, liver or kidney damage), Pb(II) (kidney damage, reduced neural development, behavioral disorders), Hg(II) (kidney damage, nervous system).

According to the scientific reports analyzed, it is concluded that there are two main risk factors in public health: (i) the intake of contaminated water, being the main factor due to direct exposure to the contaminant, which can produce different anomalies as those described in the previous paragraph. However, the studies presented cannot be considered conclusive, since the reports show that the impact on health is directly related to the clinical history of the exposed population [20]. (ii) The consumption of contaminated food, such as in the case of the report of da-Silva et al. (2019) [24], which reported Hg migration in water from the Western Amazon Basin (Amazon Triple Frontier: Brazil, Peru, and Colombia) to fish; being that if they are intended for human consumption, this can cause mercury intoxication (mercurialism). While the intake of contaminated food is the most likely action to occur, there are other special factors that particularly attract attention, such as the report presented by Oliveira et al. (2021) [87] studying a potential health risk in terms of a cognitive deficit due to soil intake by pre-school children aged 1 to 4 years, which presents high levels of Pb and Cd due to contact with contaminated wastewater from industries in the region of São Paulo (Brazil).

On the other hand, for organic contaminants, data analysis and comparison has been performed in different countries evidencing the necessity of establishing strategies to remediate water pollution (Figure 1). These strategies are urgent, based on the potential risk that these contaminants can have on human health [88,89,90]. Although there are currently certain reports, guidance values or standards that allow establishing criteria based on the presence of these contaminants and their potential toxic effect are needed [43,91]. Efforts have been performed to establish international regulations since the majority of organic compounds are not quality controls [92].

For this reason, several research groups have tried to determine the impact a chemical compound has on human health. For example, atrazine, an artificial herbicide that was detected in surface water, has been associated with an impact on human health and aquatic biota [93], upon evaluating endocrine-disrupting compounds that can affect human health via cell-based assays [94]. Moreover, per and polyfluoroalkyl substances have been determined, but there are no reference points that establish a water quality criterion for its impact on human health [91]. Based on this, there is a need to establish scientific studies in a human population and evaluate the impact of water pollution on its health. Some studies have been performed (see Table 3) to correlate the exposure of contaminants in people’s life and if possible, establish the impact that water sources and body contamination have.

### 2.3. Water Treatment Technologies for the Removal of Contaminants in Water: Status and Perspectives

#### 2.3.1. Inorganic Contaminants

Taking into consideration the environmental and public health risk represented by effluents and water bodies contaminated with metals, numerous research groups have focused on proposing remediation alternatives, highlighting the adsorption process [104,105], coagulation/flocculation [106], chemical precipitation [107], ion exchange [108], electrochemical treatments [109,110], membrane use (ultrafiltration, osmosis, and nanofiltration) [111,112], and other alternative treatments based on the use of biopolyelectrolytes and coupled adsorption processes with electrochemical regeneration [113,114]. In all cases, the actual challenge consists of evaluating the scale-up process, for which studies have been performed on a small scale under controlled conditions.

Although, scientific reports have demonstrated great efficiencies in the removal of heavy metals, there has been certain problems documented for each technology, which must be addressed to present advanced remediation technologies. For the ion exchange process, it has been documented that those present with low efficiencies for the removal of high concentrations of metals [115]. For example, Malik et al. (2019) reported removal efficiencies of 55% for Pb and 30–40% for Hg [116]. In the case of membrane filtration, good removal efficiencies have been reported (around 90% for Cu and Cd) [116],;however, it requires high installation costs and maintenance [117]. Likewise, it has been reported that the electrochemical, catalysis, and coagulation/flocculation processes present high metal removal efficiencies (around 85–99% for Cd, Zn, and Mn) [118]. On the other hand, the main drawbacks are high installation costs and extra operational costs, as well as the generation of unwanted by-products (sludge) [119]. These drawbacks significantly reduce the effectiveness of water treatment processes, so a second challenge to deal with is process optimization.

Finally, the third challenge is the design of environmentally and economically sustainable treatment processes. The current paradigm of water treatment of metal contamination must be broken; the importance is not only in water sanitation, but also in recovering the metal in order to obtain valuable products and not only change the pollutant phase [120]. For all the above, adsorption and chemical precipitation have turned out to be the most used methods. However, the removal results obtained depend on each matrix used, so the materials and experimental conditions must be proposed based on the needs and the type of effluent to be treated [121].

#### 2.3.2. Organic Contaminants

In the previous sections, the detection of these pollutants is only the first step to evaluate the environmental risk that communities and countries have in their respective water sources. The next step is to determine technologies that can establish an efficiency in the removal of these contaminants in a complex matrix without affecting the environment using novel systems [122,123,124]. In this regard, an actual challenge is the development of technologies capable of treating specific organic compounds and if it is possible, to use these treatment technologies with the current systems that governments have implemented. Some technologies that have been investigated are the use of continuous flow supercritical water (SCW) for the removal of hormones from the wastewater of a pharmaceutical industry. In their results, the technology was demonstrated to reduce 88.4% of the initial total organic carbon (TOC) value, and the presence in gas phase of H_2_, CH_4_, CO, CO_2_, C_2_H_6_, and C_2_H_4_, which could be used to produce renewable energy. Moreover, phytotoxicity assays demonstrated that there was no risk of the treated samples with respect to the germination of *Cucumis sativus* seeds [125]. Another technology that has been used is direct contact membrane distillation, which can be used to treat raw surface water contaminated with phenolic compounds [126]. In this case, water samples were spiked with 15 phenolic compounds. An important parameter evaluated was the recovery rate (RR) to demonstrate the stability of the direct membrane distillation, being up to a 30%. Pollutant removal reached 94.3 ± 1.9% and 95.0 ± 2.2% for 30% and 70% RR, respectively. A consideration for this technology is to work at a recovery rate in which flux does not decay (RR < 30%) to avoid performing loss and fouling.

Different approaches have been used for the removal of contaminants, such as the use of a photocatalytic paint based on TiO_2_ nanoparticles and acrylate-based photopolymer resin for the removal of dyes in different water matrices [127]. Another strategy was subsurface horizontal flow-constructed wetlands (planted in polyculture and unplanted) as secondary domestic wastewater treatment to demonstrate the removal of personal care and pharmaceutical products [128].

Considering the above mentioned content, among all technologies evaluated currently to eliminate organic contaminants present in water, Advanced Oxidation Processes (AOPs) stand out, since they generate highly reactive and non-selective radicals capable of almost completely mineralizing the contaminant of interest, generating mainly CO_2_ and H_2_O as an oxidation product. In this sense, the most widely studied AOPs correspond to catalytic wet peroxide oxidation, catalytic wet air oxidation, homogeneous catalyst, photo-Fenton, Fenton process, photocatalysis, Fenton-like, electro-Fenton, heterogeneous catalyst, ultrasound, and microwave [129]. Although the results show the potential use of technologies for water treatment, there are still challenges to address. The current challenge of this technology must be aimed at scaling the process, optimizing operational parameters, and designing a sustainable technology to have a low cost and be environmentally friendly, achieving the lowest generation of by-products. In this sense, two recently published research articles stand out in which AOPs have been evaluated for the treatment of contaminated water effluents in the Latin American region. Mejía-Morales et al. (2020) [130] presented the use of an AOP based on UV/H_2_O_2_/O_3_ for the remediation of residual water from a hospital in Puebla (Mexico), showing the feasibility of its use to remediate effluents contaminated with a high organic load. On the other hand, Zárate-Guzmán et al. (2021) [131] presented the scale-up of a Fenton and Photo-Fenton process for the treatment of piggery wastewater in Guanajuato (Mexico). The results show that these two AOPs have great application potential for the remediation of effluents contaminated with a high organic load due to their high removal percentages (COD, TOC, and Color) and low operating costs.

## 3. Conclusions

The presence of contaminants in the water is a severe environmental and public health problem in the American continent. The presence of inorganic (As, Cd, Cr, Pb, Cu, Hg, and U) and organic pollutants (dyes, phenolic compounds, hormones, pesticides, and pharmaceuticals compounds) in effluents and water bodies is due to anthropogenic activities and natural factors in the region. The health risks associated with these contaminants primarily encompass skin damage, carcinogenic effects, nervous system damage, circulatory system issues, kidney damage, gastrointestinal damage, and impacts on the food chain. The critical review of the reports presented in this document identifies the following as the main challenges:

(i) Implement advanced analytical detection techniques, such as those based on electrochemical tests, to achieve improvements in detection limits, low operating costs, short analysis times, and mobility to perform in situ determinations.
(ii) Accurately determine the source of contamination in each geographic site of interest to propose containment and sanitation actions to solve the problem.
(iii) Evaluate water treatment technologies on a large scale and under real conditions to optimize the treatment processes.
(iv) Design and/or conditioning of specific water treatment plants according to the pollutant of interest in the region. The universal design paradigm of a water treatment plant must be broken; the pertinent modifications must be made according to the needs of the population of interest.
(v) Design environmentally and economically sustainable treatment processes. Future water treatment processes will need to integrate circular economy concepts to obtain high-quality water and valuable products, such as precious metals, and/or produce biofuels.

## Figures and Tables

**Table 2:** Detection of organic pollutants in environmental samples.

Analyte	Samples	Region	Environmental Risk Assessment	Analytical Technique	Ref.
PCBs and PDBEs	Sediments, water, and fish	Lake Chapala (Mexico)	Bioaccumulation	GC-MS/SIM	[55]
Pesticides (herbicides, fungicides, and insecticides), and its degradates	Groundwater	USA	Carcinogens	LC-MS/MS	[67]
Inorganic (As, U, and Pb) and organic (disinfection by-products, per/polyfluoroalkyl substances, pesticides, and others)	Tapwater, untreated lake water, and treated water treatment plants	Lake Michigan (USA)	Potential risk of contamination exposure (carcinogenic)	Not specified	[68]
Pharmaceuticals, pesticides, and metals/metalloids	Surface water	Lake Guaiba (Brazil)	High toxicity in algae and aquatic invertebrates	LC-QTOF-MS, GC-MS/MS, and ICP-MS	[69]
Pesticides (antifungals, herbicides, and insecticides)	Drinking water treatment plants, public water, and sewage sites	Porto Alegre, (Brazil)	Endocrine disruption and antimicrobial resistance	SPE with LC-MS/MS system (HPLC-ESI-MS)	[70]
Antibiotics	Surface water, sediment, and natural river biofilm	Córdoba (Argentina)	Antimicrobial resistance	UPLC-ESI-MS/MS	[71]
p-Toluendiamine, p-aminophenol, and Bandrowski’s base derivative	Raw river water, drinking water, and wastewater from beauty salon	Araraquara, São José do Rio Preto in São Paulo State (Brazil)	Mutagenicity	HPLC-DAD and linear voltammetry techniques	[72]
Veterinary antibiotics	Water, sediment, and trout tissue	Lake Titicaca (Peru)	Toxic risk for algal species inhibiting protein synthesis	SPE-LC-MS/MS system	[73]
Pesticides, antibiotics, pharmaceuticals, personal care products, plasticizers, sweeteners, drug metabolites, stimulants, and illegal drugs	Pacu fillets from supermarkets and fish markets	Argentina	Potential toxicological risk in humans	Four extraction methods, two based on SPE and two on QuEChERS. Ultra-high-performance liquid chromatography coupled to a Q-Exactive Orbitrap mass spectrometer	[74]
Pharmaceutical, personal care products, PFAs, pesticides, sweeteners, stimulants	Surface water and sediments	Lake Huron to Lake Erie corridor (USA)	Endocrine disruption, cancer, antimicrobial resistance	SPE-LC-MS-MS	[75]
482 organic and 19 inorganic elements	Tap water	11 states of USA	Potential of human health risk	12 target organic and 1 inorganic methods	[76]
Polycyclic aromatic hydrocarbons, pesticides, (PCBs), and metals (Hg, Cd, Cu, Pb, Ni, Zn, and Se)	Water, sediment, and biota	Puerto Rico	Potential human health (bioaccumulation)	GC-MS, ICP-AES, CVAA	[77]
Pharmaceutical, personal care products, and pesticides	Sediments, surface, and cave water	Northern Colorado Plateau, (USA)	Potential effects in environment	LC-MS/MS with thermospray ionization, SPE-HPLC-MS/MS, GC-MS	[78]
Pharmaceutical, herbicides, and disinfectants	Untreated water ponds, wastewater reclamation sites, untreated tidal blackish rivers, non-tidal freshwater creeks, produce processing water plant (wash water)	USA	Potential human health risks	UPLC-MS/MS	[79]
Pharmaceuticals	Groundwater	Central Pennsylvania (USA)	Potential minimum human health risk	High-resolution accurate mass (HRAM), Q Exactive Orbitrap mass spectrometer through a heated electrospray injection (HESI) source	[80]
Pharmaceuticals	Raw untreated water and drinking water treatment plants	Minas Gerais (Brazil)	Presence after still treatment remains as a potential health risk	HPLC-MS	[81]
Antibiotics	Market fish	Argentina	Residues in fish can impact human health, such as antimicrobial resistance	UPLC-MS/MS	[82]
Atrazine	Synthetic and real wastewater	USA	Carcinogen	HPLC-DAD	[83]
Pharmaceuticals	Surface, wastewater, and drinking water	Canada	Elevated human risk associated with the mixture of these organic compounds	Q-TRAP LC/MS/MS	[17]
Microplastics	Wastewater	Montevideo (Uruguay)	Not mentioned	Confocal Raman Microscopy, polarized light optical microscopy, NIR spectroscopy and Scanning electron Microscopy (SEM)	[84]
Pharmaceutically active compounds	Surface and treated water (composite samples) from drinking water treatment plants	Brazil	Potential human health risk	HPLC coupled to micrOTOF-QII mass spectrometer with an ESI source	[85]
Pesticides	Water sources (rivers, lakes, lagoons, and streams)	Basin of Rio San Francisco in Minas Gerais state and urban lagoons of Belo Horizonte (Brazil)	Association with several disorders and diseases	Passive sampling device with carbon nanomaterial and GC/MS	[86]

**Table 3:** Scientific studies on the correlation between a water source and the presence of certain pollutants in a human population.

Analyte	Population	Sample	Region	Source	Analytical Technique	Ref.
Mercury and persistent organic pollutants	287 urban anglers	Blood and urine	Detroit River (USA)	Consumption of local fish	GC-ECD, ICP-MS, and HRGC/ID-HRMS	[95]
Metals and persistent organic pollutants	409 licensed anglers and 206 Burmese refugees	Blood and urine	Buffalo River, Niagara River, Eighteenmile Creek, and the Rochester Embayment	Locally caught fish, store-bought fish, and consuming fish/shellfish	ICP-MS and GC-HRMS	[96]
Al, As, Cd, Co, Cu, Hg, Mn, Ni, Pb, Se, and Zn	300 volunteers	Blood	Three regions of Brazil	Well and tapwater intake, fish, seafood consumption, and drinking water	ICP-MS	[97]
Hg, As, and Cr	32 children	Water (drinking and cooking), blood, and urine	Yucatan (Mexico)	Water source (drinking and cooking water)	(AAS) and graphite furnace AAS	[98]
B	177 mother–child cohort	Maternal blood and urine (during and after pregnancy), placenta, breast milk, infant (urine and blood), and drinking water	Argentina	Water source	ICP-MS	[99]
Fe, Pb, and Zn	353 early school-aged children	Blood, urine, and drinking water	Montevideo (Uruguay)	Not possible to establish drinking water as a main source of exposure	ICP-MS	[100]
Cd	469 people	Blood	Vila de Beja and Bairro Industrial (Brazil)	Drinking water source (general network)	ICP-MS	[101]
Nitrates	348,250 singleton births	Historical data	Missouri (USA)	Drinking water	Historical data	[25]
Pb and Cd	2433 preschoolers aged between 1 and 4-years-old	Nails	Sao Paulo, (Brazil)	Industrial activity	ICP-MS	[87]
As, Cd, Cr, Cu, Ni, Mn, and Pb	6,267,905 adults and children	Statistical data	Joanes River in the northeast of Brazil	Industrial activity	Mathematical calculation	[28]
Cd	Not specified	Blood samples	Barcarena and Abaetetuba city (Brazil)	Industry	Seronorm^®^ Trace Elements in Whole Blood Lyophilized Level 1 and Level 2 (SERO)	[101]
U, As, As, Hg, Pb, Cd, monomethylarsonic acid, dimethylarsinic acid, and Mn	231 pregnant women between 14 and 45 years of age	Blood and urine	USA	Unregulated water sources	ICP-MS (ICP-DRC-MS)	[102]
PFAS	213 non-smoking adults	Serum	USA	Home water district and bottled water	SPE-HPLC-MS/MS	[103]

## Data Availability

Not applicable.

## Supplementary Materials

The following supporting information can be downloaded at: https://www.mdpi.com/article/10.3390/ijerph20054499/s1, Supplementary Table S1: Comparative table of analytical techniques most used for the detection of inorganic contaminants present in water. Supplementary Table S2: Comparison of detection limits in μg L^−1^ at 3 sigma [132].
