# Supplementary material for: A Current Review of Water Pollutants in American Continent: Trends and Perspectives in Detection, Health Risks, and Treatment Technologies
Source: Int J Environ Res Public Health. 2023 Mar 3;20(5):4499. doi: 10.3390/ijerph20054499 (PMC10001968; doi:10.3390/ijerph20054499)
Supplement: Supplementary file 1 [file ijerph-20-04499-s001.zip › ijerph-2242483-supplementary.pdf]

International Journal of
Environmental Research and Public Health

MDPI

Review

A current review of water pollutants in American continent: Trends and perspectives in detection, health risks, and treatment technologies

Walter M. Warren-Vega, Armando Campos-Rodríguez, Ana I. Zárate-Guzmán*, Luis A. Romero-Cano*

Grupo de Investigación en Materiales y Fenómenos de Superficie. Facultad de Ciencias Químicas. Universidad Autónoma de Guadalajara. Av. Patria 1201, C.P. 45129. Zapopan, Jalisco. MEXICO.

* Correspondence: aizg_08@hotmail.com (A.I.Z.-G.); luis.cano@edu.uag.mx (L.A.R.-C.)

Supplementary Table S1. Comparative table of analytical techniques most used for the detection of inorganic contaminants present in water.

	ICP (OES & MS)	AA	Electrochemistry
Detection limits	Excellent	Very Good	Very Good
Sample throughtput	75 elements/6 min	1 element/15 s	1 element/30 s
Precision	1.0 – 3.0 %	0.1 – 1.0 %	0.1 – 1.0 %
No. of elements	75	68	Depends on electrode
Sample usage	Low	Very low	Low
Routine operation	easy	easy	Easy
Method development	Skill required	easy	Sill required
Unattended operation	Yes	No	No
Analysis	sample transport to specialized laboratory	sample transport to specialized laboratory	In situ
Operating cost	High	Low	Low
Capital cost	Very high	Low	Medium

Reference: Tyler, Geoff, and J. Yvon. "ICP-OES, ICP-MS and AAS Techniques Compared." *ICP Optical Emission Spectroscopy Technical Note* 5 (1995).

Supplementary Table S2. Comparison of detection limits in mg L^{-1} at 3 sigma

Element	ICP-MS	ICP-OES	Flame AAS	GF-AAS
As	< 0.050	< 5	< 500	< 1
Al	< 0.010	< 0.05	< 50	< 0.5
Ba	< 0.005	< 0.05	< 50	< 1.5
Be	< 0.050	< 0.05	< 5	< 0.05
Bi	< 0.005	< 5	< 100	< 1
Br *	ND	< 100	ND	ND
Cd	< 0.010	< 0.05	< 5	< 0.03
Cl *	ND	< 200	ND	ND
Ce	< 0.005	< 5	< 200000	ND
Co	< 0.005	< 1	< 10	< 0.5
Cr	< 0.005	< 1	< 10	< 0.15
Cu	< 0.010	< 1	< 5	< 0.5
Gd	< 0.005	< 5	< 4000	ND
Ho	< 0.005	< 1	< 80	ND
I	ND	< 10	ND	ND
In	< 0.010	< 20	< 80	< 0.5
La	< 0.005	< 0.05	< 4000	ND
Li	< 0.020	< 1	< 5	< 0.5
Mn	< 0.005	< 0.1	< 5	< 0.06
Ni	< 0.005	< 1	< 20	< 0.5
Pb	< 0.005	< 5	< 20	< 0.5
Se	< 0.10	< 5	< 1000	< 1.0
Ti	< 0.010	< 5	< 40	< 1.5
U	< 0.010	< 20	< 100000	ND
Y	< 0.005	< 0.5	< 500	ND
Zn	< 0.02	< 0.5	< 2	< 0.01

* Only possible with far UV capable spectrometers (< 155nm)

ICP-MS, ICP-OES, Flame AAS: Detection limits defined with 3 standard deviations

GF-AAS: Sensitivity (0.0044 absorbance) measured with 20 mL

ND = not determined

Reference: Tyler, Geoff, and J. Yvon. "ICP-OES, ICP-MS and AAS Techniques Compared." *ICP Optical Emission Spectroscopy Technical Note* 5 (1995).
